# Isolation of *Penicillium citrinum* from Roots of *Clerodendron cyrtophyllum* and Application in Biosynthesis of Aglycone Isoflavones from Soybean Waste Fermentation

**DOI:** 10.3390/foods8110554

**Published:** 2019-11-06

**Authors:** Duy Tien Doan, Duc Phuong Luu, Thanh Duong Nguyen, Bich Hoang Thi, Hong Minh Pham Thi, Huu Nghi Do, Van Huyen Luu, The Dan Pham, Van Thai Than, Hai Ha Pham Thi, Minh Quan Pham, Quoc Toan Tran

**Affiliations:** 1 Institute of Chemistry, Vietnam Academy of Science and Technology, 18 Hoang Quoc Viet St., Cau Giay Dist., Hanoi 10072, Vietnam; doanduytien@yahoo.com (D.T.D.); luuphuongtheday@gmail.com (D.P.L.); ntduong182@gmail.com (T.D.N.); 2 Institute of Natural Products Chemistry, Vietnam Academy of Science and Technology,18 Hoang Quoc Viet St., Cau Giay Dist., Hanoi 10072, Vietnam; bichhoang.inpc@gmail.com (B.H.T.); minhhcsh@gmail.com (H.M.P.T.); dohnghi@gmail.com (H.N.D.); danpt.bi9064@st.usth.edu.vn (T.D.P.); minhquanaries@gmail.com (M.Q.P.); 3 Graduate University of Science and Technology, Vietnam Academy of Science and Technology, Hanoi 10072, Vietnam; 4 Hanoi University of Natural Resources and Environment, Hanoi 10072, Vietnam; lvhuyen@hunre.edu.vn; 5 Vietnam Academy of Science and Technology, University of Science and Technology of Hanoi, Hanoi 10072, Vietnam; 6 NTT Institute of High Technology, Nguyen Tat Thanh University, Ho Chi Minh City 700000, Vietnam; tvthai@ntt.edu.vn; 7 Faculty of Biotechnology, Nguyen Tat Thanh University, Ho Chi Minh City 700000, Vietnam

**Keywords:** *Penicillium citrinum*, β-glucosidase, soybean extract, fermentation, isoflavones, aglycones

## Abstract

Soybeans offer an abundant source of isoflavones, which confer useful bioactivities when existing in aglycone forms. The conversion of isoflavones into aglycones via fermentation of soybean products is often realized by β-glucosidase, an enzyme produced by fungi. In this study, a filamentous fungus, *Clerodendron cyrtophyllum*, was isolated from root of *Clerodendron cyrtophyllum* Turcz, which was able to produce the highest activity of β-glucosidase up to 33.72 U/mL at 144 h during fermentation on Potato Dextrose Broth (PDB). The obtained fungus was grown on isoflavones-rich soybean extract to produce genistein and daidzein, achieving the conversion rate of 98.7%. Genistein and daidzein were isolated and purified by column chromatography using hexane/acetone (29:1/1:1), reaching purities of over 90% of total isoflavones, as identified and determined by TLC, LC-MS/MS, and ^1^H and ^13^C NMR spectroscopy. These results imply that the isolated *P. citrinum* is a potential fungal strain for industrial-scale production of genistein and daidzein from isoflavones-containing soybean extracts. These products may serve as potential raw materials for manufacture of functional foods that are based on aglycones.

## 1. Introduction

Isoflavones are polyphenolics that exert estrogen-like effects and have been widely utilized in manufacture of foods and cosmetics [1]. Isoflavones exhibit a myriad of bioactivities including protecting against colon cancer and aging skin, easing postmenopausal symtoms in women, reducing the risk of osteoporosis, preventing cardiovascular disease, having antimutagenic, antioxidant, and anti-inflammatory effects, and acting as tyrosine kinase enzyme inhibitors [1,2]. Since the intestinal absorption of isoflavone in aglycone form, particularly geistein, is more expedited than in the glycosylated form, most beneficial biological properties have been suggested to originate from aglycone isoflavone [2]. However, the content of aglycones is significantly lower than that of the glycoside counterpart, accounting for only 5% of total isoflavones. Structurally, the phenolic group of isoflavones is bonded to glycosides and primary hydroxyl group of glucose moiety is bonded to 6-O-acetyl or 6-O-malonyl derivatives. It has been shown that isoflavones existed in 12 isomers: three free isoflavones (aglycones) and nine linked isoflavones (glucosides) [3,4,5,6].

In traditional Asian diets, the main supply of isoflavones is from soybean (*Glycine max*) [7]. Soybean contains a large amount of bioactive subtances such as isoflavones, saponin, phytosterol, protease inhibitor, inositol hexaphosphate, and trypsin inhibitors [8]. As the bioavailability of soy isoflavone aglycones is superior to that of the glycoside forms, the isolation of isoflavone aglycones from soy products as well as production of aglycones for pharmaceutical and functional food applications have attracted research interest recently [9,10].

Industrial production of soybean oil generates byproducts such as soapstocks and soy cakes. While the former, which is rich in fatty acids [11], could be utilized in production of animal feeds, soap, nonpetroleum waxes, and biofuels, the latter was recently suggested to play an important part in the biotechnological production of several high-added value chemicals, such as fumaric acid by fungi, carotenoids by yeasts, and polyol esters and wax esters by the enzymatic synthesis [12,13,14]. The production of such soybean-derived products are realized via various techniques including saponification [15], esterification, and enzymatic hydrolysis [16].

The common production technique of aglycones involves hydrolysis of isoflavone glucosides using chemical catalysts (e.g., bases or acids) [8]. Alternatively, the transformation of isoflavones glycosides to aglycones can be also achieved during soybean germination as a natural occurence or by selective enzymatic hydrolysis and fermentation with β-glucosidase-producing microorganisms [17,18,19,20,21]. The latter has attracted considerable attention by researchers as a green approach.

β-Glucosidase (β β -D-glucoside glucohydrolase; EC 3.2.1.21), an exocellulase responsible for the hydrolysis of the O-glycosyl linkage of terminal nonreducing β-D-glucosyl residues releasing β-D-glucose, e.g., the bond in cellobiose, has been the enzyme of interest in large scale production via fermentation with microbes [22,23]. To be specific, feasible microbes that have been utilized in β-glucosidase production include *Aspergillus* niger [24,25], *A. oryzae* [26], *Penicillium* brasilianum [27], *Phanerochaete chrysosporium* [28], *Aspergillus* oryzae [29], *Thermofilum pendens* [30], yeasts (mostly *Candida* sp.) and several bacterial species. Even though fermentation to produce β-glucosidase for hydrolysis using *A. niger*, a filamentous fungus, is popular and has been the standard in commercial β-glucosidase production [31], emerging approaches utilize various strains from the *Penicillium* genus [27]. These new techniques also produce lipase and esterase, which act as catalysts for hydrolysis of ester groups of 6-O-acetyl and 6-O-malonyl isoflavones derivatives for activation of hydrolysis of glycosides by β-glucosidase.

In this work, multiple β-glucosidases-producing fungi were isolated and screened from roots of the *Clerodendron cyrtophyllum* Turcz plant. The fungal strain that exhibited the highest β-glucosidase activity was selected and then used for fermentation of isoflavones-rich soybean extract to produce aglycones, genistein, and daidzein. The products were identified and determined by thin layer chromatography (TLC), liquid chromatography-tandem mass spectrometry (LC-MS/MS), and nuclear magnetic resonance (NMR).

## 2. Materials and Methods

### 2.1. Materials

Sabouraud-2% dextrose Broth (SDB), Sabouraud-4% glucose-Agar (SA), potato dextrose broth (PDB), potato dextrose agar (PDA), p-nitrophenyl-β-d-glucopyranoside (pNPG), p-nitrophenol (pNP), and methylene blue were purchased from Merck KGaA (Darmstadt, Germany). Roots of *Clerodendron cyrtophyllum* Turcz, locally collected at Hoai Duc ward, Hanoi, Vietnam, were coarsely ground to the size of 5 mm and then were divided equally to two parts. One part was subjected to sun drying and sterilization using UV light for 10 min while the other part was stored in gunny bags. The sized *C. cyrtophyllum* Turcz’s roots were used for isolation of the β-glucosidase-producing fungi. Oil-extracted soybean residue (dry form) (*Glycine max* L.) was purchased from T&H Agriculture and Technology, JSC (Hanoi, Vietnam).

### 2.2. Isolation of Microorganisms

Fungal strains were isolated by serial dilution [32]. Briefly, 1 g of the sized *C. cyrtophyllum* Turcz’s roots was mixed with 10 mL of sterile deionized water. The following serial dilution was performed to 10^−5^ and 0.1 mL of the suspension was dispersed onto the sterilized PDA agar plates, which were allowed to stand for 72 h at 30 °C to promote fungal growth. Different types of fungi were isolated. They were then subcultured on sterilized PDA plates several times to obtain pure cultures, which were maintained at 4 °C for further study.

### 2.3. Fungal Identification

The fungal cultures were obtained by repeatedly transferring onto sterilized PDA plates and inoculated at 30 °C for 72 h. After inoculation, characteristics such as color and size of colonies during the growth was monitored and recorded. A little amount of mycelial mat was placed on clean glass slide and then stained with methylene blue. These slides were microscopically analyzed for morphological characteristics. Fungal isolates were identified by comparing these characteristics with those listed in standard reference book [33].

To prepare DNA, each fungal strain was cultured and incubated on PDA plates for three days at 30 °C. Purelink Genomic DNA Kits (Life Technology, ThermoFisher Scientific, Massachusetts, USA) was used to obtain DNA from the hyphae of the isolate. Two primer pairs, ITS1 (5′-TCCGTAGGTGAACCTGCGG-3′) and ITS4 (5′-TCCTCCGCTTATTTGATATGC-3′) were used to perform the amplification of the the internal transcribed spacer region (ITS) of the nuclear ribosomal DNA operon [34]. PCR experiments were performed as follows. First, a 20 µL mixture containing 5–50 ng of DNA, AccuPrep PCR premix (Bioneer, Daejeon, South Korea), and 5 pmol of each primer was created. The mixture was first denatured at 95 °C for 5 min. Afterwards, a total of 30 cycles of denaturation, annealing, and extension took place with the elapsed time of 30 s in each cycle. Temperature for the three processes was 95, 48, and 72 °C, respectively. The protocol concluded with extension at 72 °C for 7 min. Electrophoretic characterization of the obtained PCR products was performed in 1% agarose gel. Obtained genomic sequences were referenced to GenBank via BLAST search tool (http://blast.ncbi.nlm.nih.gov/Blast.cgi) and were found to be in line with reported sequences.

### 2.4. Enzymatic Activity

Fungal strains were maintained on PDA at 30 °C with periodic transfer of five days. Inoculum was prepared by growing the fungal mycelium in 100 mL sterilized basal medium, which consisted of 2 g/L (NH_4_)_2_SO_4_, 1 g/L KH_2_PO_4_, 0.4 g/L MgSO_4_·7H_2_O, and 0.5 g/L CaCl_2_ (pH = 5.6) and 10% (*w*/*v*) PDB as carbon source in 250 mL Erlenmeyer conical flasks. Incubation was carried out at 120 rpm using a rotary shaker, at 30 °C for 72 h. After incubation, β-glucosidases produced by different fungi strains in flasks were extracted by supplementing with potassium phosphate buffer 20 mM (pH = 6), followed by shaking at 200 rpm for 1 h and then filted by using membrane Supro 450 size 0.45 um (Pall, Ann Arbor, MI, USA). The obtained filtrates were used as crude enzymes. The hydrolysis of p-nitrophenyl-β-d-glucopyranoside (pNPβG) substrate was taken as the measure for β-glucosidase activity of fungi [35]. The reaction mixture contained 30 µL of appropriately aliquot (the filtrate) from incubated flask, 60 µL of pNPβG and 210 µL of acetate buffer. The reaction commenced at 50 °C for 60 min, followed by addition of 300 µL of 1 M Na_2_CO_3_. The resultant release of p-nitrophenol (pNP) caused color change, which was measured spectrophotometrically at 410 nm. The enzyme quantity required to maintain the pNP release rate of 1 µmol per minute is equivalent to one unit of β-glucosidase activity.

### 2.5. Optimization of the β-Glucosidase Production

The incubation of *Penicillium citrinum* was carried out in a 250 mL Erlenmeyer flask containing 100 mL of described basal medium under rotary shaking at 120 rpm at 30 °C for seven days. β-glucosidase activity was determined at different intervals starting from the beginning of incubation, including 24, 48, 72, 96, 120, 144, and 168 h, using the aforementioned procedure.

Effect of carbon sources on growth and enzyme production were evaluated by the following procedure. First, 1 mL aliquot of SDP-grown *Penicillium* sp. was placed into a 250 mL Erlenmeyer flasks containing 100 mL the basal medium with adding 0.75% (NH_4_)_2_SO_4_, 50 mM KH_2_PO_4_. Afterwards, different carbon sources including either PDB, corn, potato, barley, and Czapek’s Dox (Cz) were supplemented at the concentration of 3% (*w*/*v*). Incubation of flasks was carried out using a rotary shaker (120 rpm) at 30 °C for the optimum time obtained in the previous section.

### 2.6. Fermentation of Soybean Residual Using P. citrinium

First, 50 g of soybean residue collected after the extraction process of soybean oil, accruing from the extraction process of soybean oil, was used as a feedstock for isoflavones extraction by using 100 mL of ethanol/water (70/30, *v*/*v*) and 70 °C for 2 h. The extracted mixture was filtered to obtain filtrate. The extraction experiment was repeated three times and the filtrates were pooled together before ethanol removal by vacuum distillation to obtain the final extract (designated as CDN) containing concentrated isoflavones for fermentation experiments. The fermentation mixture was made up by mixing 20% (*w*/*v*) glucose and 5–20% (*v*/*v*) of CDN extract, 0.75% (*w*/*v*) (NH_4_)_2_SO_4_, 50 mM KH_2_PO_4_, pH 4.5 followed by sterilization at 121 °C for 20 min. A fungal amount of 2% (*v*/*v*) of *P. citrinium* was used to inoculate the medium prepared in 250 mL Erlenmeyer flasks and incubation at 30 °C for 120 h with shaking rate of 180 rpm. Samples were regularly taken at 24, 48, 72, 96, and 120 h of fermentation, followed by addition of ethanol, vortexing, and centrifugation to obtain supernatants for the determination of the conversion of glucosides, genistin, and daidzin into aglycones and corresponding glycosides.

Hydrolysis rate was calculated by the following equation:

$$H=\frac{\mathrm{isoflavone}\;\mathrm{glycosides}\;\mathrm{in}\;\mathrm{control}\;\mathrm{samples}-\mathrm{isoflavone}\;\mathrm{glycosides}\;\mathrm{in}\;\mathrm{hydrolyzed}\;\mathrm{samples}}{\mathrm{isoflavone}\;\mathrm{glycosides}\;\mathrm{in}\;\mathrm{control}\;\mathrm{samples}}\times 100$$

Isoflavone aglycones in the hydrolyzed mixture were isolated and purified by means of column chromatography (Mini-C, diameter of 70 mm) on silica gel (Merck 60, 15–40 µm, height loaded of 10 cm) using hexane/acetone (29:1/1:1).

### 2.7. Determination of Glucosides and Aglycones Using TLC, LC-MS/MS, NMR

To perform TLC (thin layer chromatography) analysis, silica gel 60 F_254_ plates were used. The employed developing solvent was a mixture of chloroform/methanol/water (80:20:2, *v*/*v*/*v*, lower phase). The revelation of spots were performed by spraying with FeCl_3_ 5% or 1% Ce(SO_4_)_2_/H_2_SO_4_, followed by exposure to UV lamp with wavelength of 254 nm till spots were clearly observed. Identification of glucosides and aglycones was made by comparison with reference standards. An LC instrument (Agilent 1100 system, Santa Clara, CA, USA) was employed to perform LC analysis at a detection wavelength of 260 nm, the injection volume of 20 µL with the reverse phase C25 ODS-2 (250 mm × 4.6 mm, 5 µm) column. Two solvents, A (acetonitrile 100%) and B (formic acid 0.15%), were used as the mobile phase. The gradient conditions were as follows. [100–50% B, 0–50 min]; [50–20% B, 50–52 min]; [20–0% B, 52–60 min] with a flow rate of 1.0 mL/min. Mass spectra of Electron Spray Ionisation (ESI) were recorded on the Agilent 1100 Series mass spectrometer connected with Varian 320-MS (LabWrench, Midland, Canada). ^1^H-NMR spectra were recorded by 500 MHz (Bruker XL-500, Billerica, MA, USA) with DMSO-d_6_ or acetone-d_6_ as solvents and tetramethylsilane as an internal standard. ^13^C-NMR spectra were recorded at 125 MHz (Bruker XL-500) with DMSO-d_6_ or acetone-d_6_ as solvents and as internal standard.

### 2.8. Statistical Anaylsis

Experiments were carried out in triplicate for the accuracy of data. Statistical significant differences were realized at *p* < 0.05 via Student’s *t*-test. Statistical analysis was performed in the JMP Pro 13.2 software.

## 3. Results

### 3.1. Isolation and Screening of β-Glucosidase-Producing Microorganisms

Roots from *Clerodendron cyrtophyllum* Turcz sp. were used as microbial sources to isolate fungi with β-glucosidase activity on PDA medium. At 10^−5^ dilution, colonies were isolated separately. They were then subcultured on sterilized PDA plates several times to obtain pure culture for identification and β-glucosidase enzyme assay. After several subcultures at 30 °C for 72 h, 10 fungi were isolated with different morphological characteristics of colony. Five fungi were isolated from fresh samples (designated as BC) of *C. cyrtophyllum* Turcz’s while five other fungi were isolated from dried plant samples (designated as BSD).

**Figure 1:**
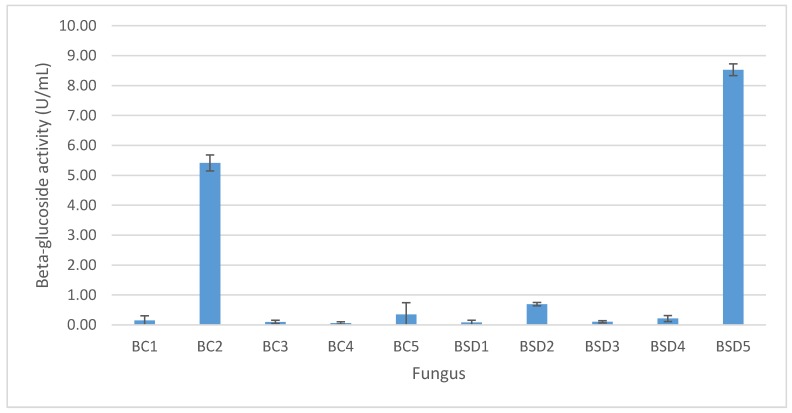
β-glucosidase activity of the isolated fungi.

For enzymatic screening and fungal selection, the ten fungal isolates were tested for their capability of enzyme production. The β-glucosidase activity of these fungi is shown in the Figure 1. The strains BC2 and BSD5 were determined as the most potential fungi for enzyme production (5.41 ± 0.27 and 8.53 ± 0.20 U/mL, respectively) in comparison with other strains (<0.63 U/mL) and were therefore selected for further studies.

### 3.2. Isolation and Screening of β-Glucosidase-Producing Microorganisms

On SA medium, colonies of BC2 grew fast and displayed a compact green or yellow basal felt enclosed by a layer of white of erect conidiophores. The diameter of colonies was approximately measured as 2.4 cm. Microscopically, conidiophore stipes are long and smooth-walled and the color of hyaline turned dark towards the vesicle. Conidial heads were biseriate, large, globose, and showed tendency to radiate, splitting into several loose columns with age. Additionally, phialides, usually in the form of septate metulae, were also observed. These morphological characteristics affirm that this fungus belongs to the *Aspergillus* species.

The shade of colonies of BSD5 growing on SA medium was green in color and a dense felt of conidiophores was observed. The average diameter of colonies after three days of incubation was 1.2 cm. Observing under a microscope, conidiophores were hyaline and smooth-walled. In addition, terminal verticils, carrying 3–5 metulae each, were observed on conidiophores. In each metula, around 3–7 phialides were recognized. Regarding conidia, they are smooth-walled and their shape appeared to be globose or subglobose. In addition, the production of conidia was basipetal from the phialides. Therefore, the described fungus belonged to the *Penicillium* species.

**Figure 2:**
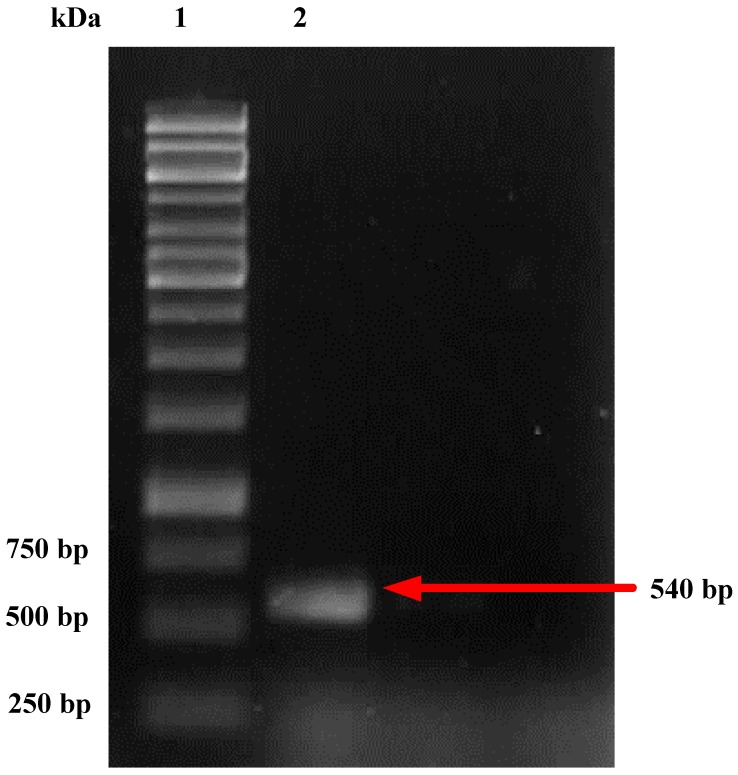
PCR amplification of genomic DNA of *P. citrinum* (lane 2) on agarose gel 1%. Lane 1—DNA size marker.

After identification by morphological characteristics, genomic DNA of the *Penicillium* sp. was identified to ensure that this fungus belonged to *Penicillium* sp. Figure 2 shows the PCR products on agarose gel 1% in which lane 1 is marker proteins and lane 2 is DNA of the fungus. It can be demonstrated that the molecular mass of this fungus is 540 bp. By using ITS method, the genome DNA of the *Penicillium* sp. was sequenced as:

“CATGCTCCGGCCGCCATGGCGGCCGCGGGAATTCGATTTCCGTAGGTGAACCTGCGGAAGGATCATTACCGAGTGCGGGCCCCTCGGGGCCCAACCTCCCACCCGTGTTGCCCGAACCTATGTTGCCTCGGCGGGCCCCGCGCCCGCCGACGGCCCCCCTGAACGCTGTCTGAAGTTGCAGTCTGAGACCTATAACGAAATTAGTTAAAACTTTCAACAACGGATCTCTTGGTTCCGGCATCGATGAAGAACGCAGCGAAATGCGATAACTAATGTGAATTGCAGAATTCAGTGAATCATCGAGTCTTTGAACGCACATTGCGCCCTCTGGTATTCCGGAGGGCATGCCTGTCCGAGCGTCATTGCTGCCCTCAAGCCCGGCTTGTGTGTTGGGCCCCGTCCCCCCCGCCGGGGGGACGGGCCCGAAAGGCAGCGGCGGCACCGCGTCCGGTCCTCGAGCGTATGGGGCTTCGTCACCCGCTCTAGTAGGCCCGGCCGGCGCCAGCCGACCCCCAACCTTTAATTATCTCAGGTTGACCT”.

To gain insights into the evolutionary relationship, two methods for creation of the phylogenetic trees, namely Neighbor Joining (NJ) and Maximum Parsimony (MP), were employed, resulting in almost identical topologies. The aligned dataset consisted of 42 taxa and 100 characters. Our sequence (marked as unknown) achieved 100% matching with those of *Penicillium citrinum* existing in the database, as evidenced by the excellent bootstrap results. Therefore, the examined fungus presumably belonged to the *Penicillium* genus and firmly aligned with the *P. citrinum* species, as demonstrated by the the strong sequence similarities with the said species.

### 3.3. Optimization of β-Glucosidase Production by P. citrinum

**Figure 3:**
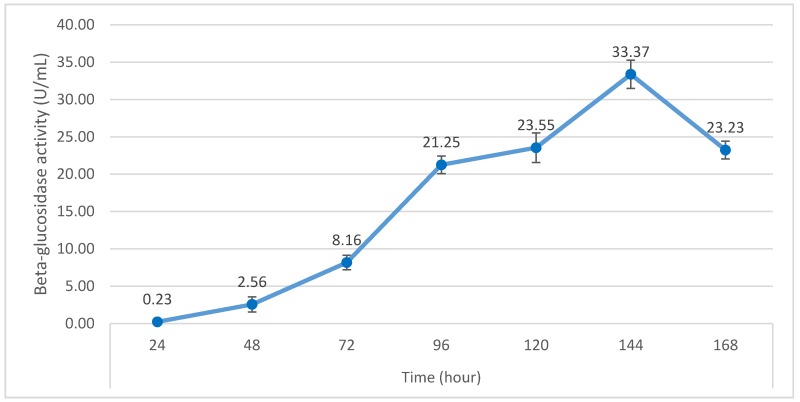
Effect of fermentation time on β-glucosidase activity of *P. citrinum*.

The optimum incubation time is the time interval at which the highest β-glusosidase activity was attained. After incubation for 24, 48, 72, 96, 120, 144, and 168 h, β-glusosidase activity was 0.23, 2.56, 8.16, 21.25, 23.55, 33.37, and 23.23 U/mL (Figure 3), respectively. This indicates that the highest β-glucosidase activity of 33.63 U/mL was achieved at 144 h of the fermentation time and that prolonged incubation time seemed to reduce enzyme yield. This could be explained by the reduced quantities of nutrients, in both micro and macro forms, existing in the medium after an extended period of fermentation. The phenomenon highlights the role of fungal physiology in inactivating secretary machinery of the enzymes. In addition, the rapid enzyme production in the initial period might be attributable to the high resistance to microbial hydrolysis of the materials, which was later diminished as the fermentation time elapsed.

**Figure 4:**
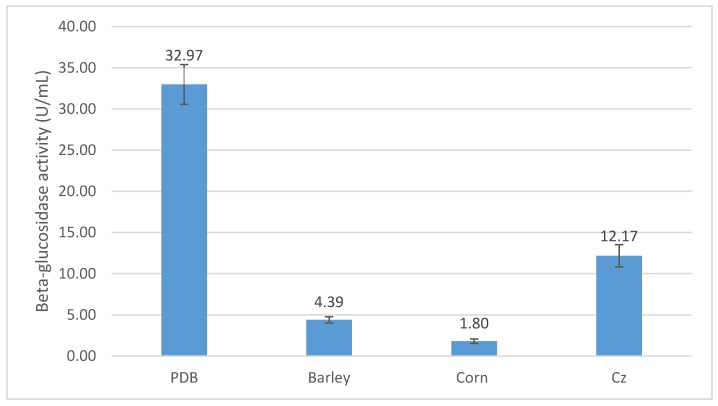
Effect of different carbon sources on β-glucosidase activity of *P. citrinum*.

Carbon source is a critical factor affecting the production of enzymes. Therefore, *P. citrinum* was tested to grow in fermentation medium containing various carbon sources under the temperature of 30 °C, agitation speed of 200 rpm, and for six days. Figure 4 shows that the highest β-glucosidase activity of 32.97 U/mL was achieved on potato, and the lower β-glucosidase activities of 4.39, 1.80, and 12.17 U/mL were recorded on barley, corn, and Cz sources, respectively.

### 3.4. Fermentation of Soybean Residual Extract by P. citrinum for Aglycones (Genistein, Daidzein) Production

**Figure 5:**
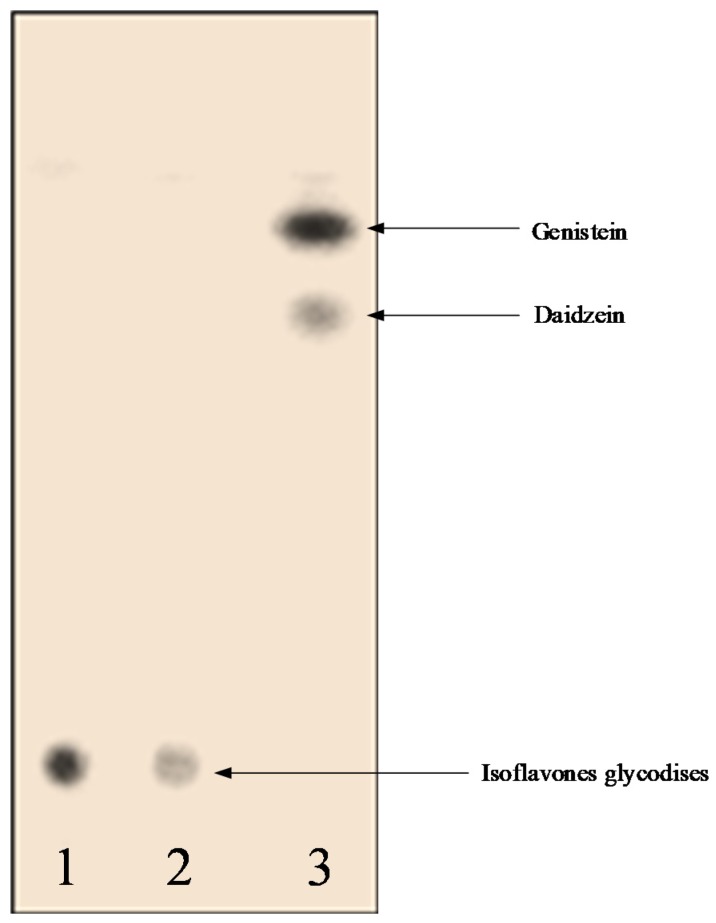
TLC results of fermentation of soybean residual extract using *P. citrinium* at 30 °C during (1) 2 h, (2) 48 h, and (3) 72 h.

Figure 5 and Table 1 show that soybean residual extract originally contained glucosides, which were still dominant in the first 24 h fermentation, but partly hydrolyzed after 48 h and completely converted to aglycones after 72 h fermentation. Moreover, the data revealed that at 10–20% (*v*/*v*) volumetric ratio of *P. citrinum* inoculum to CDN substrate, the transformation into aglycones from glucoside forms complete took place during 72 h of fermentation (Table 2). This indicates that the optimal conditions for fermentation of soybean residual extract to aglycones was 10% (*v*/*v*) of *P. citrinum* enzyme to CDN with fermentation time of 72 h at 30 °C.

### 3.5. Identification and Characterization of Aglycones Using LC-MS/MS, TLC, and NMR

**Figure 6:**
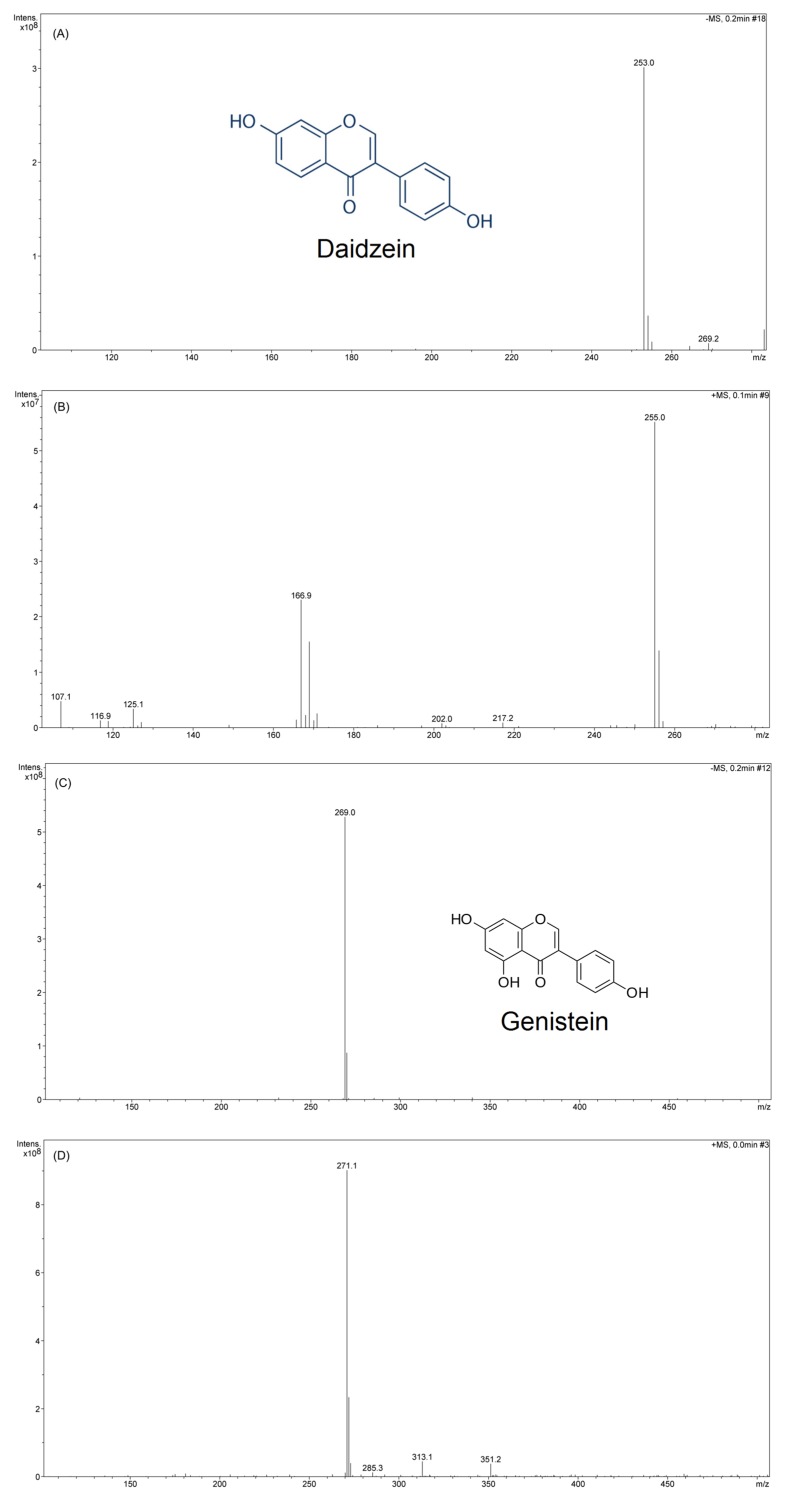
LC-MS/MS spectra of daidzein and genistein. Negative mode of daidzein at m/z 253 (**A**), positive mode of daidzein at m/z 255 (**B**), negative mode of genistein at m/z 269.0 (**C**), and positive mode of genistein at m/z 271.1 (**D**).

LC-MS/MS results reveal that in the first stage of fragmentation in the negative ion mode, one ion of high intensity at m/z 253 (Figure 6A) was observed, which corresponded to the elemental composition of (C_15_H_10_O_4_, daidzein) with molecular weight of 254.23 (Figure 6B). On the other hand, in the positive ion mode, the highest intensity fragment appeared at m/z 255, corresponding to the same ion having an elemental chemical composition of (C_15_H_10_O_4_). Data indicates that daidzein obtained after extraction and purification from fermentation broth is of high purity. Similarly, genistein ion fragments appeared at m/z 269.0 (Figure 6C) and m/z 271.1 (Figure 6D), having the highest intensity in the negative and positive mode, respectively. These findings are in line with the results of the standard reagents and of previously reported works [3,7].

For genistein (DTE1, 99.80 mg, purity 90%), light yellow needle crystals, R_f_ = 0.37 (TLC, silica gel, solvent n-hexan/acetone (7/3, *v*/*v*)) appeared as yellow-brown with 5% FeCl_3_ and dark green with Ce(SO_4_)_2_ (data not shown). ^1^H-NMR (Acetone, 500 Hz): δ (ppm) 6.28 (1H, d, J = 2 Hz, H6), 6.41 (1H, d, J = 2.5 Hz, H8), 6.90 (2H, dd, J = 2 Hz, H3′, H5′), 7.46 (2H, dd, J = 2 Hz, H2′, H6′), 8.163 (1H, d, J = 1.5 Hz, H2), 13.03 (1H, s, OH). ^13^C-NMR (Acetone, 125 Hz): δ (ppm) 94.15 (s, C8), 99.52 (s, C6), 105.87 (s, C10), 115.65 (d, C3′, C5′), 122.75 (s, C1′), 123.76 (d, C3), 130.86 (d, C2′, C6′), 153.98 (s, C2), 163.64 (d, C5), 158.76 (d, C9), 158.13 (t, C4), 164.70 (t, C7), 181.35 (t, C7).

For daidzein (DTE2, 300.58 mg, purity 90%); white needle crystals, R_f_ = 0.30 (TLC, silica gel, solvent n-hexan/acetone (7/3, *v*/*v*)) appeared as dark-green with Ce(SO_4_)_2_ and colorless with 5% FeCl_3_ (data not shown). ^1^H-NMR (Acetone, 500 Hz): δ (ppm) 6.89 (3H, dd, J = 2 Hz, 9 Hz, H3′, H5′, OH4′), 6.98 (1H, m, H8), 7.47 (2H, dd, J = 5.5 Hz, 2 Hz, H2′, H6′), 8.06 (1H, d, J = 8.5 Hz, H6), 8.14 (1H, s, H2). ^13^C-NMR (DMSO, 125 Hz): δ (ppm) 102.04 (s, C8), 115.07 (d, C6), 116.58 (d, C5′, C3′), 123.43 (d, C3), 127.23 (t, C1′), 129.99 (d, C5), 125.74 (s, C2), 157.11 (d, C4′), 157.37 (d, C9), 162.45 (t, C7), 174.63 (s, C4).

## 4. Discussion

It was reported that β-glucosidase can be produced on different carbon sources by fungi with different activities [36]. Lachke et al. [37] found that the maximum β-glucosidase activity of *Penicillium funiculosum* was 30–36 U/mL, achieved on 3% rice bran or defatted oil cakes after 288 h fermentation. By stark contrast, the peak activity was found at a much lower level of 2.8 U/mL with *Penicillium miczynskii* cultured on 3% pineapple peel within 216 h of fermentation (Table 3) [38]. Furthermore, the investigation of Jeya et al. [39] resulted in the highest β-glucosidase activity of 26.4 U/mL in *P. purpurogenum* produced on rice straw, while Ng et al. [40] achieved the highest β-glucosidase activity of 57.5 U/g solid when rice bran was used via solid fermentation. Although other variables (e.g., temperature, pH, etc.) have not been optimized in this study, β-glucosidase produced by the isolated *P. citrinum* was reached 33.63–33.72 U/mL, listing *P. citrinum* as the top fungal strain among the reported fungi producing highest activity of β-glucosidase. In terms of scalability, β-glucosidase holds the potential to be cloned into other hosts such as *Escherichia coli* for large-scale industrial production [41].

The hydrolysis yield of soybean waste extract (rich in isoflavone glycosides) to their aglycones by *P. citrinum* was estimated to be 98.7%, which is comparable to aglycone rates of 94.22 and 97.14% achieved by *D. hansenii* UFV-1 immobilized cells containing β-glycosidase and free enzyme, respectively [42], on soy molasses. However, our reported hydrolysis rate is higher than the rate of 93% achieved in deglycosylation of extracts of soybean flour and embryo in β-glucosidase derived from *Paecilomyces thermophila* J18 [43]. Moreover, the biotransformation rate is also higher than that by a marine *Streptomyces* sp. 060524, which achieved only 90% on the same substrate during 108 h fermentation [5].

Isoflavone aglycones can be derived from soybean waste by extraction with ethanol/water solvent followed by acid hydrolysis and purification [44], reaching purity of 92%. Alternatively, the soybean extract can be hydrolyzed by cellulase to produce crude aglycone isoflavones, followed by purification, to obtain purity of 80.38–87.21%. Experimental data obtained in this study showed that the purity of genistein and daidzein reached over 90%, which is comparable to the purity obtained by the reported methods, demonstrating raw materials with potential for utilization in industrial applications, e.g., pharmaceutics and functional foods, with minimum refining [3].

## 5. Conclusions

The *P. citrinum* fungus strain that produces the highest β-glucosidase activity of 33.72 U/mL, thus placing it amongst the most active fungi in this regard, has been successfully isolated from roots of *Clerodendron cyrtophyllum* Turcz. The fungus demonstrated a catalytic capacity to hydrolyze isoflavones-rich soybean extract into aglycones (e.g., genistein and daidzein) with a hydrolysis yield of 98.7% after 72 h of fermentation at 30 °C. Purification of the hydrolyzed mixture (rich in genistein and daidzein) by column chromatography using hexane/acetone (29:1/1:1) resulted in aglycone products with purity of over 90%. These results imply that soybean extract is a promising raw material for manufacture of functional foods derived from aglycones. Our study demonstrated a potential pathway for the production of aglycones from residual soybean extract via fermentation with an isolated fungi as a biocatalyst, with applciations in the pharmaceutical and functional food industries.

## Figures and Tables

**Table 1:** Effect of hydrolysis time of soybean residual extract on isoflavon glucosides and aglycones formation by *P. citrinium* at 30 °C.

Fermentation Time (h)	Isoflavone Glucosides	Aglycones	Remark
0	++++	-	Original soybean extract without addition of enzyme
24	++++	-	
48	+++	+	
72	-	++++	
96	-	++++	
120	-	++++	

Note: ++++, dark stain; +++, medium stain; +, weak stain; -, no stain.

**Table 2:** Effect of volumetric ratio of soybean residual extract/enzyme on isoflavone glucosides and aglycones formation by *P. citrinium* at 30 °C.

Soybean Residual Extract/Enzyme Ratio (*v*/*v*, %)	Isoflavone Glucosides	Aglycones	Remark
0	++++	-	Original soybean extract without addition of enzyme
5	-	+	
10	-	+++	
15	+	+++	
20	++	+++	

Note: ++++, dark strain; +++, medium stain; ++, medium-weak stain; +, weak stain; -, no stain.

**Table 3:** β-glucosidase production of several fungal strains.

Fungal Strain	Fermentation Conditions	β-Glucosidase Activity (U/mL)	Ref.
*P. funiculosum*	Substrate: Rice bran, defatted oil cakes Temperature: 20 °C pH: 4.5 Fermentation time: 288 h	30–36	[34]
*P. miczynskii*	Substrate: Pineapple peel Temperature: 30 °C pH: 5.5 Fermentation time: 216 h	2.82	[35]
*P. purpurogenum* KJS506	Substrate: Rice straw Temperature: 32 °C pH: 4 Fermentation time: 144 h	26.4	[36]
*P. citrinium* YS40-5	Substrate: Rice bran Temperature: 70 °C pH: 6.0 Fermentation time: 96 h	57.5 U/g	[37]
*P. citrinium*	Substrate: PDB Temperature: 30 °C pH: 4.5 Fermentation time: 144 h	33.72	Current study
